# Editorial: Therapeutic potential of innate and innate-like effector lymphocytes in autoimmune and inflammatory diseases

**DOI:** 10.3389/fimmu.2023.1323486

**Published:** 2023-10-31

**Authors:** Lan Wu, Fu-Dong Shi, Luc Van Kaer

**Affiliations:** ^1^ Department of Pathology, Microbiology and Immunology, Vanderbilt University School of Medicine, Nashville, TN, United States; ^2^ Department of Neurology, Tianjin Medical University General Hospital, Tianjin, China; ^3^ Center for Neurological Diseases, China National Clinical Research Center for Neurological Diseases, Beijing Tiantan Hospital, Capital Medical University, Beijing, China

**Keywords:** immunotherapy, innate lymphocytes, innate-like lymphocytes, innate lymphoid cells, mucosal associated invariant T cells, natural killer cells, natural killer T cells

Modulating the immune system holds great promise for treating a variety of autoimmune and inflammatory diseases, by stimulating desired immune responses and/or by inhibiting undesired immune responses. Traditional therapeutic modalities for autoimmune and inflammatory conditions exert global suppressive effects on immune responses and often impair overall immune competence, as they quell both pathogenic and protective immune responses. More recently developed biological therapies selectively suppress the pathogenic responses in autoimmune and inflammatory diseases by acting on specific immune cell subsets or the inflammatory mediators they produce. The latter treatments require a thorough understanding of the underlying disease pathology, especially the role of antigen-specific effector B and T lymphocytes of the adaptive immune system. However, comparatively little attention has been paid to the immunotherapeutic potential of innate and innate-like effector lymphocytes, which also make important contributions to the development and progression of autoimmune and inflammatory diseases. This Research Topic focuses on our emerging understanding of the roles of innate and innate-like lymphocytes in the pathogenesis of autoimmune and inflammatory diseases, and how this information might be exploited for the development of new and improved immunotherapies.

Innate and innate-like lymphocytes share a number of features that make them particularly attractive targets for immunotherapy. For example, their specificity is not impacted by polymorphic major histocompatibility complex (MHC) ligands and, therefore, uniform tools could be employed to elicit their therapeutic properties in genetically disparate individuals. Furthermore, their therapeutic activation or inactivation might not lead to widespread immune impairment and susceptibility to infection or cancer.

Among the innate lymphocytes, natural killer (NK) cells have been studied predominantly for their anti-tumor and anti-viral activities. Yet, these cells also play critical roles in a variety of other diseases. Wang et al. review the controversial role of NK cells in sepsis, where they might contribute to protective immune responses against the invading microbes, but also to the overall hyper-inflammatory phase of sepsis by producing cytokines and causing tissue destruction, and to the subsequent immune-suppressive phase of sepsis where they might adopt a hyporesponsive phenotype rendering the host susceptible to secondary infection. The original research article by Qi et al. explores the features of NK cells in patients with Alzheimer’s disease, revealing quantitative and qualitative alterations in these cells, and the presence of a unique NK cell subset whose prevalence negatively correlates with patient cognitive functions. Although NK cells are best known for displaying innate effector functions, in some situations, such as during infection by human cytomegalovirus (HCMV), some NK cells might exert adaptive-like memory responses. The activating NKG2C receptor on such NK cells might engage with HCMV-derived peptides bound with the unconventional human leukocyte antigen (HLA)-E protein. The primary research article by Almazán et al. describes the identification of three peptides from HCMV that induce such NK cell-mediated memory responses. The investigators also generated synthetic versions of these HCMV peptides that could be potentially employed as therapeutic vaccines.

In addition to NK cells that were discovered nearly fifty years ago, work during the past two decades has identified multiple additional innate lymphocyte subsets. This growing family of innate lymphoid cells (ILCs) is typically partitioned into three groups: group 1 includes NK cells and ILC1 cells producing type 1 cytokines such as interferon (IFN)-γ, group 2 includes ILC2 cells producing type 2 cytokines such as interleukin (IL)-4 and IL-5, and group 3 includes ILC3 cells and lymphoid tissue inducer cells producing type 3 cytokines such as IL-17. Jia et al. review the contributions of distinct ILC subsets to the development of atopic dermatitis, with a focus on the pathogenic role of ILC2s. Zhang et al. discuss the controversial role of group 3 ILCs in intestinal diseases, which might be related to the capacity of these cells to respond to dietary metabolites and gut microbiota. Thomas and Peebles review IL-10-producing ILCs, which have been observed among all ILC subsets. These cells display a regulatory phenotype that promotes gut and lung homeostasis, with the potential for therapeutic applications in intestinal and lung diseases.

A unique subset of innate lymphocytes with T cell features and with the capacity for IFN-γ production and cytotoxicity has been identified within the intestinal epithelium. Hariss et al. present new findings on these so-called innate intestinal intraepithelial lymphocytes (iIELs) during infection by the intestinal, protozoon pathogen *Cryptosporidium parvum*, revealing the capacity of these iIELs to control parasite proliferation at early stages of the infection.

Multiple subsets of T lymphocytes display innate-like functions, including CD1d-restricted natural killer T (NKT) cells, MHC class I related-1 (MR1)-restricted mucosal-associated invariant T (MAIT) cells, innate subsets of T cell receptor (TCR) γδ-expressing T cells, and subsets of innate-like, TCR-expressing iIELs. Bharadwaj and Gumperz review different ways by which the anti-inflammatory properties of NKT cells might be harnessed to control pathological inflammation, and how differences in the functional properties between murine and human NKT cells will likely make this goal rather challenging. Lee et al. review the role of NKT cells to immunity in the skin, where these cells can exert either protective or pathogenic effects on inflammatory skin diseases, raising the possibility to modulate NKT cell effector functions for immunotherapy. The original research article by Imahashi et al. also focuses on skin inflammation, using a mouse model of allergic contact dermatitis, to explore the role of MR1-restricted MAIT cells to disease, showing that these cells are activated quickly following challenge with a contact allergen to suppress skin inflammation. Joyce et al. review the contribution of NKT cells and MAIT cells to a wide variety of infectious agents, arguing that these cells can integrate signals delivered by innate sensor cells responding to pathogens and then relay those signals to downstream innate and adaptive immune effector cells. Finally, Zhou reviews the role of NK cells, NKT cells, MAIT cells, and γδ T cells to early liver inflammation in various contexts, how such cells might trigger chronic liver inflammation and fibrosis, and how their depletion might be able to attenuate several liver diseases.

Similar to the T cell lineage, subsets of B lineage cells with innate-like functions have been identified. This includes marginal zone B (MZB) cells that reside at the interface between the circulation and lymphoid tissue, and B-1 B cells that reside primarily in the mesothelial cavities. Tandel et al. review yet another B cell subset, referred to as natural killer-like B (NKB) cells, with reported innate-like characteristics. While some research groups have confirmed that NKB cells express characteristic markers of both NK cells and B cells, other researchers have argued that these cells display phenotypic and functional characteristics of conventional B cells, prompting the need for further investigations of their origin and identity.

The articles included in this Research Topic provide elegant examples of the exciting, ongoing work on innate and innate-like lymphocytes, highlighting the potential of targeting these cells for immunotherapy of human autoimmune and inflammatory diseases.

## Author contributions

LW: Writing – review & editing. FS: Writing – review & editing. LVK: Writing – original draft.

